# Transposable Elements Are a Major Cause of Somatic Polymorphism in *Vitis vinifera L.*

**DOI:** 10.1371/journal.pone.0032973

**Published:** 2012-03-12

**Authors:** Grégory Carrier, Loïc Le Cunff, Alexis Dereeper, Delphine Legrand, François Sabot, Olivier Bouchez, Laurent Audeguin, Jean-Michel Boursiquot, Patrice This

**Affiliations:** 1 UMT Geno-Vigne®, IFV-INRA-Montpellier SupAgro, Montpellier, France; 2 UMR AGAP, INRA Montpellier, Montpellier, France; 3 UMR DIADE, IRD-UM2-CIRAD, Montpellier, France; 4 Plateforme GénoToul, INRA Auzeville, Castanet-Tolosan, France; Kyushu Institute of Technology, Japan

## Abstract

Through multiple vegetative propagation cycles, clones accumulate mutations in somatic cells that are at the origin of clonal phenotypic diversity in grape. Clonal diversity provided clones such as Cabernet-Sauvignon N°470, Chardonnay N° 548 and Pinot noir N° 777 which all produce wines of superior quality. The economic impact of clonal selection is therefore very high: since approx. 95% of the grapevines produced in French nurseries originate from the French clonal selection. In this study we provide the first broad description of polymorphism in different clones of a single grapevine cultivar, Pinot noir, in the context of vegetative propagation. Genome sequencing was performed using 454 GS-FLX methodology without *a priori*, in order to identify and quantify for the first time molecular polymorphisms responsible for clonal variability in grapevine. New generation sequencing (NGS) was used to compare a large portion of the genome of three Pinot noir clones selected for their phenotypic differences. Reads obtained with NGS and the sequence of Pinot noir ENTAV-INRA® 115 sequenced by Velasco *et al.*, were aligned on the PN40024 reference sequence. We then searched for molecular polymorphism between clones. Three types of polymorphism (SNPs, Indels, mobile elements) were found but insertion polymorphism generated by mobile elements of many families displayed the highest mutational event with respect to clonal variation. Mobile elements inducing insertion polymorphism in the genome of Pinot noir were identified and classified and a list is presented in this study as potential markers for the study of clonal variation. Among these, the dynamic of four mobile elements with a high polymorphism level were analyzed and insertion polymorphism was confirmed in all the Pinot clones registered in France.

## Introduction

Genomes were thought to be stable constituents of living organisms until Barbara McClintock's discovery of genome plasticity opened up a new avenue of research [1]. Dynamics of genomes have thus become an important field of research, SNPs and short indels being the most widely studied polymorphisms. These have a potential impact on phenotypic variations [2], in particular non-synonymous SNPs located in regulatory regions [3], [4]. Similarly, mobile elements drive genome evolution [5], playing an important role in mutations responsible for genomic reorganizations [6] and genome size variations [7]. In this way, 82% of the maize genome is composed of overlapping mobile elements [8]. Other mechanisms of genome regulation such as epigenetic variations [9], [10] chromosome rearrangements [11] and copy number variations [12], [13] could also have an impact on phenotypic variations.

A significant number of domesticated plants including banana, potato, grape, coffee tree are vegetatively propagated to maintain agronomically valuable genotypes [14]. However, after many propagation cycles, clones accumulate phenotypic differences in agronomic traits and clonal diversity appears [15]. This diversity can then be used to select the best clones within a given variety. Indeed, several clonal selection programs for grape, potato or banana have led to the release of new certified clones with very significant gains for the industry. In particular, clonal diversity in grape is used to select the best clones for commercial purpose as it is the only solution to access a plant diversity without modifying the identity of cultivars with worldwide repute. Cultivar identity is crucial in the case of appellation wines in Europe which are produced from a restricted list of specific cultivars.

Vegetative propagation has been used since the end of Antiquity period [16] and allows grape to display a remarkable clonal diversity [17]. Previous studies of grapevine clonal diversity using SSR markers enabled the identification of limited clonal polymorphism in a few groups of clones [18], [19]. However SSR analyses are not an efficient way to distinguish genetic differences between clones [20], [21]. Alternatively, the S-SAP approach using universal retrotransposon based primers revealed polymorphism between five Pinot clones [22] although use of *Vine-1* based primers [23] failed to reveal any variation between six Pinot clones [24]. Pinot is one of the oldest grape cultivars [25], [26] and among the noblest, being used notably in Champagne and Bourgogne wines. It displays extensive clonal diversity and, in France alone, 64 different Pinot clones are certified and marketed [26]. Furthermore Pinot noir was the cultivar choosen in grapevine genome sequencing projects: the grape reference genome using a near homozygous line PN4024 [27] derived from Pinot Noir cultivar by successive selfings and the second sequencing project using Pinot noir clone ENTAV-INRA® 115 (PN115) [28]. Pinot studies can now fully benefit from existing genomic tools since the release of the reference genome sequences [27], [28] available through the grape genome browser (http://www.genoscope.cns.fr/)

New generation sequencing (NGS) has changed the landscape of genetics and genomics studies and allowed questions to be answered at genome scale [29], [30]. Until now, no study has proposed a broad description of polymorphism linked to vegetative propagation. In the present study, we thus exploited the power of NGS and the grape genomic tools to perform a genome-wide comparison of grape clone genomes without *a priori* knowledge. In order to quantify the different types of polymorphisms (SNP, indel, mobile elements) likely involved in clonal diversity, we sequenced 3 Pinot noir clones (PN386, PN583, PN777) selected for their phenotypic differences using 454 GS FLX methodology. We compared a portion of these Pinot noir clones with the available sequences of PN115 [28] after alignment on the PN40024 reference genome. Consequences of theses polymorphisms will be discussed as well as potential uses of these results for the wine industry.

## Results

### Alignment and representation of the Pinot noir clone sequences on the reference genome

#### Genome reconstruction by alignment

We analyzed sequences of four clones of Pinot noir (PN115, PN386, PN583 and PN777) selected to maximize the phenotypic diversity of this cultivar.

PN115 sequences were downloaded from ncbi database (http://www.ncbi.nlm.nih.gov/) and correspond to published work [28]. PN386, PN583, PN777 sequences were obtained by 454 sequencing methodology. These four sets of sequences were aligned on reference sequence PN40024 [27]. For PN115 a total of 67% of the sequences were aligned with the 3 steps procedure (Table 1). They correspond to single locus regions. Since sequences matching more than one locus were discarded. For the other clones an average of 62% of reads was aligned on the PN40024 sequence (Table 1). This represent a mean coverage of 32% of PN40024 sequence at 1.00 fold genome coverage (base count) but only 0.3% at 6.00 fold genome coverage (Table 2).

**Table 1:** Description of the results of the alignments on PN40024 for the different sequenced clones by 454 methodology and for PN115 available sequences.

		PN386	PN583	PN777	Mean of 3 clones	PN115
% of aligned sequences	Alignment Step 1	48.1	42.5	40.2	43.6	57.7
	Alignment Step 2	0.9	1.1	1.0	1.0	1.3
	Alignment Step 3	12.5	21.7	16.7	16.9	8.0
	Total of aligned reads	61.5	65.3	57.9	61.5	67.0
% of unaligned sequences	Repeat elements	12.5	13.4	13.90	13.2	12.7
	Paralogs	12.0	10.1	13.0	11.7	20.3
	Cytoplasmic DNA	4.2	3.6	3.7	3.8	
	Unknown	8.1	6.8	10.4	8.4	
	Contamination (other organisms)	0.01	0.01	0.01	0.01	
	Low quality reads	1.7	0.8	1.4	1.3	
	Total of unaligned reads	38.5	34.8	42.1	38.4	33.0

Proportion of aligned reads in each steps of the alignment process and proportion of unaligned reads on the reference genome. First alignment step: aligned reads with 95% identity on single loci with reference sequence; Second alignment step: reads aligned in this step are composed by a repeat element (between 50–300 b) which was masked and by a unique sequence (greater than 150 b) which allowed alignment; Third alignment step: reads in this step are aligned on reference sequence with a gap parameter fixed at a minimum.

**Table 2:** Coverage of clones genomes.

	PN386	PN583	PN777	Common regions	Reference genome covered	PN115 genome covered
Coverage 1× or more	113 Mb	132 Mb	139 Mb	95 Mb	194 Mb	168 Mb
Coverage 2× or more	46 Mb	64 Mb	44 Mb	16 Mb	122 Mb	98 Mb
Coverage 3× or more	15 Mb	25 Mb	14 Mb	0.2 Mb	54 Mb	52 Mb
Coverage 4× or more	6 Mb	11 Mb	5 Mb	0 Mb	22 Mb	22 Mb
Coverage 5× or more	3 Mb	5 Mb	2 Mb	0 Mb	10 Mb	10 Mb
Coverage 6× or more	1.3 Mb	2.2 Mb	1 Mb	0 Mb	4.5 Mb	4.5 Mb

Size of the portion of genome aligned on the reference genome at different coverage levels for the three sequences clones and PN115. Common regions correspond between all clones sequenced in 454 GS-FLX. In the polymorphism call we only considered regions with 6.00 fold genome coverage or more.

Among unaligned sequences, only 8% of the reads did not match any known reference sequences of PN40024 (Table 1). These sequences may be either unknown repeated elements, unassembled regions of PN40024 or due to a contamination not reported in any database. The remaining unaligned reads which corresponding to paralog (12%) and repeat sequences (13%), were not retained due to multiple possible localizations on the reference sequence. Reads alignment quality was estimated using an alignment quality score (ranging from 0 to 90) [31], 90% of the aligned sequences have a quality score higher than 60 (see Supplementary Figure S1).

#### Comparison with the reference genome

**Figure 1:**
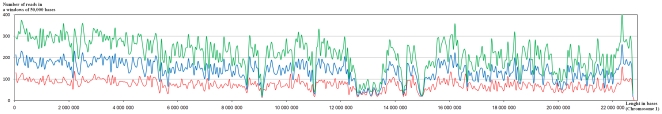
Read alignment on chromosome 1. To test the random distribution of reads, three runs were sequentially aligned. The first 454 run was aligned (red line) on chromosome 1. Then both first and second runs were aligned together (blue line), and finally all three runs (green line) were aligned on the chromosome. The insufficiently covered region around 13 Mb in chromosome 1 corresponds to the centromere.

We compared several criteria (percentage of exons, GC, CpG and CnG among the aligned sequences) between clones and PN40024 and no difference were observed (Table 3). The number of aligned bases on each chromosome was proportional to their length (R^2^>0.62, see Supplementary Figure S2). However, our results indicate that read distribution along the chromosomes was non random and some regions were consistently excluded from alignment (see Figure 1 for an example on chromosome 1). Low-alignment regions showed over-representation of repeat elements in some areas, particularly at the centromere assumed location. There is a significant negative correlation between the number of aligned sequences and the number of repeat elements annotated in the reference sequences (correlation coefficient <−0.25 and p-value<0.01).

**Table 3:** Composition of 454 reads aligned with the reference genome.

	PN386	PN583	PN777	PN40024
% GC in aligned sequences	36.0	35.0	35.0	33.0
% CpG in aligned sequences	2.4	2.4	2.4	2.2
% CnG in aligned sequences	0.9	0.9	0.9	0.9
% Exons	9.9	10.6	7.9	6.9

We compared the percentage of GC, CpG, CnG and exons in the 454 data set and the reference genome. Percentage of GC, CpG, CnG were estimated with a Perl script. Percentage of exons was estimated by Blast 2.0 (id>85%) with the annotation of the reference genome dated 19 March 2010.

### Polymorphism calling

**Figure 2:**
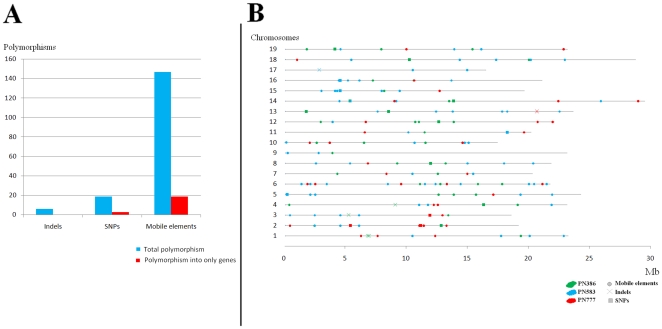
**Results** **from polymorphism call.** **A**) **Number of polymorphisms detected between, each pair of clones**; Numbers of SSR, SNP, indel and mobile element polymorphisms between each pair of clones in regions of 6.00 fold genome coverage only covering 4.5 Mb of genome. **B**) **Map of polymorphism between clone PN115 and clones PN386, PN583, PN777.** All types of polymorphisms (SNPs, indels, mobile elements) detected between PN115 and partially 454-sequenced (6.00 fold genome coverage ) clones (green, blue, red for PN386, PN583, PN777 respectively). SNPs, indels and mobile elements are represented by crosses, squares, and diamonds respectively.

**Figure 3:**
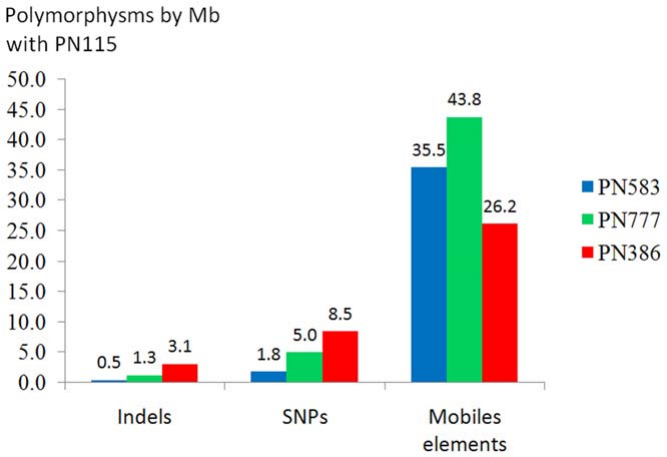
Number of SSR, SNP, indel and mobile element polymorphisms with PN115 per Mb of genome sequence for each clone. In red PN386, in blue PN583 and in green PN777.

In order to eliminate any risk of false positive polymorphism detection from clones sequenced by 454 methodology, we choose to analyze and call polymorphisms only from sequenced regions at 6.00 fold genome coverage (least 6 independent reads should be aligned at each base pair). Moreover, for the polymorphic positions the minor allele should be present in at least 30% of the independent sequences. Because of the absence of common regions between the 3 sequenced clones at 6.00 fold genome coverage we compared each sequenced clone with only PN115 which is a true clone of Pinot noir contrary to PN40024. In total, the sum of the sequences shared by one of the 3 clones and PN115 represents 4.5 Mb (around 1% of grape genome) at 6 fold genome coverage (Table 2). We detected no SSR, but 19 SNPs, 6 indels and 147 sites with a polymorphic insertion of mobile elements (Figure 2A and Supplementary Table S1) representing a mean of 1.6 (+/−1.0) SNPs, 5.1 (+/−2.7) indels and 35.2 (+/−7.2) mobile elements per Mb (Figure 3). Among these putative polymorphisms, 1 indel, 3 SNPs and 19 sites of mobile elements insertion per Mb were localized in genes (predicted from the reference genome −19 March 2010 version- ; Supplementary Table S2). Polymorphisms were well distributed throughout the genome (Figure 2B).

### Distribution and dynamics of mobile elements

#### Identification of mobile elements

In the partial sequences of clones PN386, PN583 and PN777, we searched for the different mobile elements known in grape [32], [33]. Among the 107 known mobile elements in grape, 62 have generated at least one insertion polymorphism (see Supplementary Table S3). Polymorphic elements belong to either class I (72%) or class II (23%) mobile elements. The most abundant ones in sequenced clones were LINES retrotransposons, followed by Gypsy and Copia-like elements. However, Gypsy famiy was the most elements which generate insertion polymorphisms between clones studies (Supplementary Table S3).

#### Selection of mobile elements and confirmation of their insertion polymorphism

We selected for detailed analyses four representative mobile elements among class I LTR transposable elements: *Gret-1*, *Copia-10*, *Gypsy-19* and *Cauliv-1*. These four elements have very different copy numbers and polymorphic sites in the partial sequenced of the clones: *Gret-1* displayed 64 copies with 5 polymorphic sites; *Copia-10*, 1273 copies with 4 polymorphic sites; *Gypsy-19*, 564 copies with 3 polymorphic sites and *Cauliv-1* 1065 copies with 2 polymorphic sites (Supplementary Table S3).

**Figure 4:**
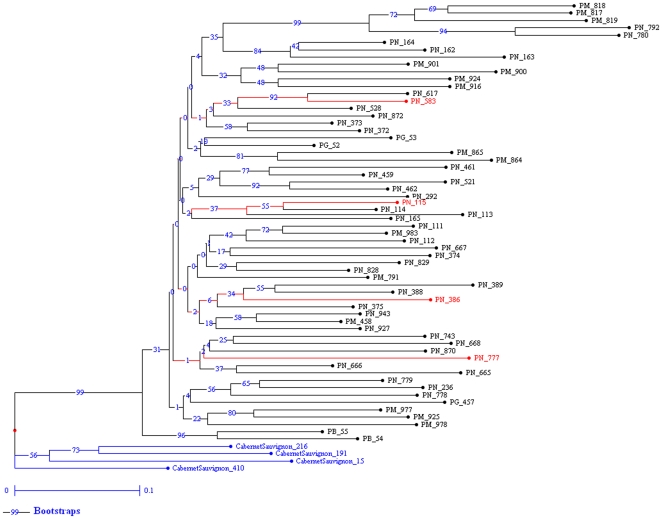
Tree of all registered Pinot clones based on S-SAP data with 4 mobile elements. S-SAP performed with *Gret-1*, *Copia-10*, *Gypsy-19* and *Cauliv-1* mobile elements. All analyzed clones have a specific pattern for these elements. 60 Pinot clones (PN = Pinot noir (40) ; PM = Pinot meunier (15) ; PG = Pinot gris (3) ; PB = Pinot blanc (2)) and 4 Cabernet-Sauvignon clones were analyzed.

To confirm polymorphism due to these mobile elements we performed a S-SAP [34] analysis based on their specific sequences on the 60 Pinot clones registered in France including PN115, PN386, PN583, PN777. We found a total of 134 polymorphic bands (37% of total scored band) among all clones and each clone displayed a specific pattern for these four elements as illustrated in the phenetic tree based on Nei and Li distance matrix [35] from presence/absence of the bands (Figure 4). For the four clones studied in detail (PN115, PN386, PN583, PN777), we found on average 45 polymorphic bands between any 2 clones (see Supplementary Table S4).

#### Dynamic of mobile elements

LTR distribution and diversity were analyzed in detail for the four mobile elements selected (*Gret-1*, *Copia-10*, *Gypsy-19* and *Cauliv-1*). First, within the entire 454 data set, we identified the major forms of consensus LTR and estimated the representation of each of their major forms in the genome (Table 5). Major forms represented by at least 10 locus with 90% identity. Four LTR consensus were identified for *Gret-1* and *Copia-10*, representing 51% and 36% of total LTRs, whereas only one consensus was identified for *Gypsy-19* and *Cauliv-1*, representing less than 10% of the total number of LTRs (Table 4). Minor LTR forms, too divergent to allow building of LTR consensus sequences, represented respectively 93%, 90%, 64% and 49% of identified LTR in *Cauliv-1*, *Gypsy-19*, *Copia-10* and *Gret-1* (Table 5).

**Table 4:** Distribution of consensus LTRs from clones sequenced by 454 methodology.

	LTR1	LTR2	LTR3	LTR4	Minor LTR
Gret-1	25%	18%	5%	3%	49%
Copia-10	19%	7%	7%	3%	64%
Gypsy-19	10%	/	/	/	90%
Cauliv-1	7%	/	/	/	93%

Reads identified as mobile element LTRs were clustered with AAARF software to build the consensus LTR. For the different mobile elements the table shows the number of consensus LTRs built and their representation in our data sets.

**Table 5:** Sequencing statistics of the raw data.

Clones	Sequencing size (Mb)	Number of reads	Mean length of reads	Mean quality of reads	Number of duplicated reads	Over-represented reads	Contaminations	
							S. cerevisiae	E coli
PN386	330	941498	351	31	12239 (1.3%)	No	25	2
PN583	378	1052396	361	28	2420 (0.23%)	No	202	2
PN777	344	988669	354	31	2642 (0.26%)	No	163	3
Mean	351	994188	355	30	4974 (0.58%)	No	130	2

**Figure 5:**
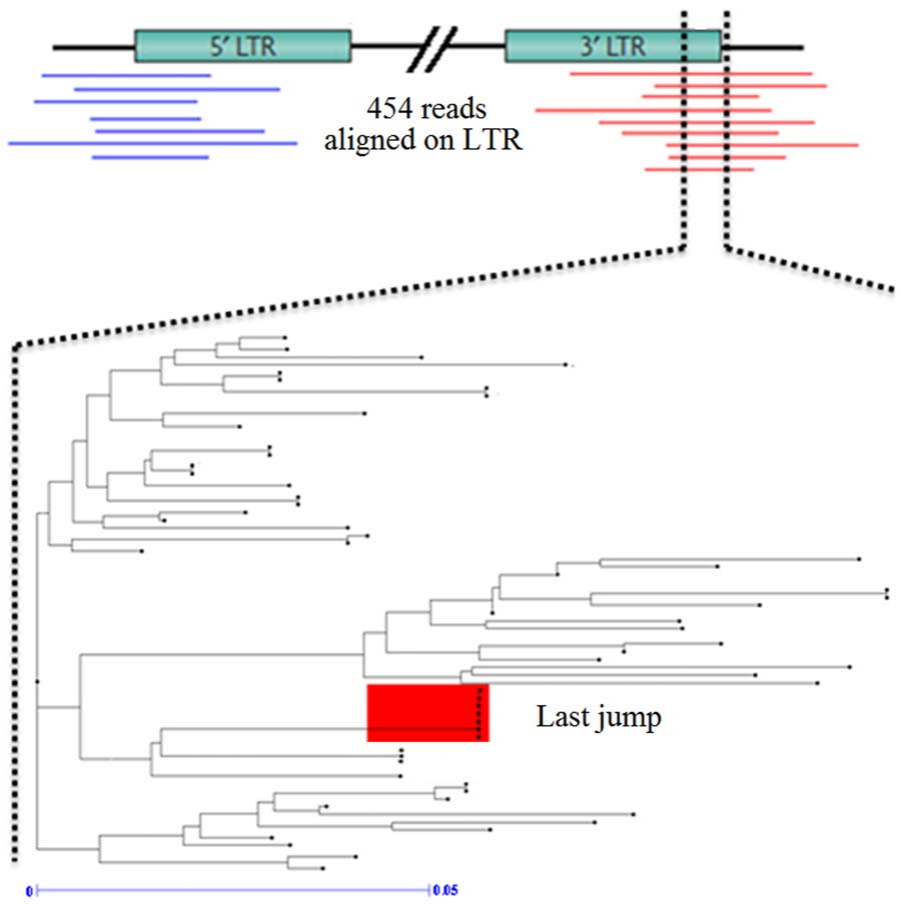
*Gret-1* tree from the consensus sequence detected in LTR. The consensus region used to build the tree for *Gret-1* mobile element is indicated by the dashed line. On the tree, the group of similar sequences (circled in red) suggests recent activity of *Gret-1*.

Then we built trees based on sequence homology using the conserved region detected in the LTR sequences of these four elements. This conserved region contains the integrase sequence in the 3′ LTR [36] (Figure 5). Results for *Gret-1* showed a typical pattern of recent activity with several copies of very homologous sequences. No such patterns were obtained for *Copia-10*, *Gypsy-19* and *Cauliv-1* (Supplementary Figures S4, S5 and S6).

## Discussion

The present work represents the first genome-wide analysis of polymorphism among grape clones without *a priori* in an attempt to identify all the molecular polymorphisms involved in somatic mutations. Four Pinot noir clones (PN115, PN386, PN583 and PN777) were selected for their distinct phenotypic characteristics (for example yield or sugar content [26]). The clonal selection was performed making prospection in old vineyards, clone PN115, PN386, PN583 and PN777 were selected in different fields in Bourgogne (France) in 1971, 1975, 1978 and 1981 respectively. At this time wood was collected from one particular plant in the field. For each clone history of this plant or of the vineyard was by consequence not available and it is impossible to date the time of divergence between clones. Interestingly, although we have revealed SNPs and indels in this study, the most important mutational events in the context of vegetative propagation were however the insertion polymorphisms generated by mobile elements. Progress in sequencing methods allowed to access to a part of the genome at a total cost and in a time span that were unachievable just a few years ago [30].

### Partial sequencing of Pinot clone genomes

We chose to work on Pinot, one of the most diverse cultivars in term of morphology. An average of 62% of the reads obtained by 454 methodology was aligned at a single locus on the reference sequence and 25% of the reads were not consider because they matched at more than one locus. Our results are similar to those obtained in *Vitis* by Myles *et al.* [37]. The grape genome is an ancient hexaploid genome [27] and has many paralogous regions that complicate mapping, particularly for short reads. This is an another reason why we preferred the 454 methodology to any other.

Clone sequenced by 454 methodology (PN386, PN583, PN777) were compared with the PN115 sequence produced by Velasco *et al* [28] which corresponds to assembly with a mean at 6.4 fold genome coverage. In order to perform this comparison, we have aligned all sequences on the reference sequence (PN40024).

The random distribution of reads obtained with the 454 method enabled access to a representative part of the grape genome. All chromosomes were covered proportionally to their length, and percentages of GC, CpG and CnG and exon composition were similar between 454 sequences and the reference genome [27]. Major parts of the chromosome regions were easily sequenced and aligned. Only regions containing many repeat elements such as centromere, telomere, and satellite regions were difficult to analyze using this re-sequencing protocol.

### Identification of dynamic events involved in somatic genome evolution

We searched for molecular polymorphism among grape clones in order to identify the most significant and dynamic elements involved in vegetative (or somatic) evolution. To limit false positives, only bases sequenced at least six times (corresponding to mean coverage depth of the PN115 sequences [28]) and with alignment quality scores higher than 60 were considered, conditions that have already been used in similar studies [38], [39], [40]. Regions shared by PN115 and at least one of the other sequenced clones at 6.00 fold genome coverage represented a total size of 4.5 Mb (approx. 1% of the genome).

Until now, previous studies of clonal diversity, mainly focused on SSRs and AFLP markers, enabled only limited identification of clones [18], [19], [20], [21]. Although they present a quite low mutation rate, both SNPs and indel have been identified in our studies and are therefore potential markers to study clonal diversity. The related polymorphism rate is however quite low, since we found 1.6 SNPs and 5.1 indels per Mb, while polymorphism between cultivars can be as high as 20 000 SNP per Mb [41]. Although they are less abundant than mobile elements, SNPs are known to generate polymorphism when they are located in genes. As an example, one SNP modification in the *VvGAI-1* gene of a Pinot meunier clone resulted in a dwarf phenotype [42]. In the present study, one SNP between PN777 and PN115, is located in one exon and generates a non-synonymous mutation (Supplementary Table S4). This candidate gene could be associated with phenotypic differences and, considering the low cost of the analysis, one can suggest that clone and/or somatic mutant sequencing might be an interesting way to identify candidate genes linked to grape polymorphism.

The major cause of somatic polymorphisms were insertion polymorphisms caused by mobile elements since 147 events were observed (35.2 per Mb). Such great extents of mobile elements polymorphism strongly suggest somaclonal transcriptional activation. Mobile elements are known to generate a substantial number of mutations that can impact gene expression and genome size, while sequence duplications can also be responsible for new gene functions [5], [36], [43]. In grape, variation of grape berry color for example was due to the insertion of the *Gret-1* element into the *VvMybA-1* promoter [44]. In our study, 19 out of 147 events involving mobile elements are found in genes. These specific elements could be used in the future with S-SAP or other protocols to study clonal diversity.

This level of polymorphism generated by mobile elements is high. Validations on other samples are presently in progress on genome wide analysis of clonal variation. It will allow comparisons with diversity at cultivar level as wel. Since no other work has been reported comparison is impossible. Nevertheless, S-SAP analysis using 4 elements (*Gret-1, Copia-10, Gypsy-19, Cauliv-1*) also revealed high insertion polymorphisms generated by mobile elements: 30% of total bands were polymorphic between clones. Moisy *et al.*, [45] studying distribution of mobile elements in 7 cultivars using S-SAP observed that 80% of the bands were polymorphic between cultivars showing high polymorphism between cultivars.

### Dynamics of mobile elements linked to vegetative multiplication

For all partially analyzed genomes, we determined the number of copies of each mobile element (Supplementary Table S3). The LINES retrotransposon family was the most widely represented (5 LINES among the 6 most abundant elements) followed by Gypsy and Copia-like elements. The same result was obtained in the reference genome, with 75% of repeat elements corresponding to LINES members [27]. Activity of Gypsy family elements is known to generate high polymorphism in plants [43] and indeed, although they were less numerous than LINES elements, Gypsy elements showed higher polymorphism than LINEs.

We analyzed LTR distribution and diversity in detail for the four mobile elements (*Gret-1*, *Copia-10*, *Gypsy-19* and *Cauliv-1*) and identified for each element several consensus LTR which could be correlated to mobile elements activity. In fact, the more frequent representation of major forms over minor forms for one element suggests a high level of recent activity. Interestingly, in our study, mobile elements ranked in the same order when classified by their percentage of major forms or by their number of polymorphism insertions, conforting analysis accuracy (Supplementary Table S3 and Table S4). *Gret-1* had the lowest proportion of minority forms and generated most of the insertion polymorphism in all partially analyzed genomes. In contrast, *Cauliv-1* had the highest proportion of minority forms and generated the lowest level of insertion polymorphism among the 4 studied elements.

Figure 5 shows the pattern displayed by *Gret-1* with similar LTR sequences that had no time to diverge. In the last years, studies have shown that *Gret-1* is a “recent” mobile element [45], [46] with reportedly recent activity since *Gret-1* insertion into the *VVMybA1* color regulating gene is believed to have occurred after grape domestication some 7000 years ago [47].

### A list of potential markers

The S-SAP approach has been used to analyse clonal diversity but with very contrasting results according to the mobile elements tested. Wegscheider *et al.* [22] used universal retrotransposon-based primers and revealed polymorphism among five Pinot clones. But Verriès *et al.*, [23] using *Vine-1* based primers, failed to reveal any variation among six Pinot clones. A wider choice of mobile elements which can be used as markers in clone diversity studies could therefore be very appropriate and the list of mobile elements presented in this paper may thus help the grapevine genetics community in the selection of efficient markers. We tested four of these elements with a high level of insertion polymorphism (*Gret-1*, *Copia-10*, *Gypsy-19* and *Cauliv-1*) in Pinot clones registered in France. Each clone displayed a specific pattern for these elements (Figure 4), thus confirming the high level of insertion polymorphism they could have generated by transposition activity. Although this was not the aim of our study, these elements might be used to study diversity in Pinot and other grape cultivars as all four Cabernet Sauvignon clones studied here *(CS15, CS191, CS216, CS416)* also displayed a specific pattern for these mobile elements (Figure 4). Caution should however be exercised in the use of S-SAP as this method might be hindered due to high mobile element activity. Markers base specific locus should therefore be preferred.

### Conclusion

Genome-wide comparison of spontaneous grape clones enabled the first study of the molecular polymorphisms generated along vegetative propagation at whole genome scale. Although a small number of SNP and indel events were also observed, mobile elements were involved in most polymorphisms. Gypsy-like elements being were the most polymorphic ones. This study identified 172 polymorphic sites in a cumulative analysis of 4.5 Mb of the grape genome, which represent a higher polymorphism level than initially expected for vegetative propagation material. Additional analyses are now underway in order to analyze a larger part of the genome of the clones already studied as well as new clones and work clones of other cultivars to confirm our results.

## Materials and Methods

### Plant material and DNA extraction

Three clones of *Vitis vinifera L.* cultivar Pinot noir n° ENTAV-INRA® 386 (PN386), 583 (PN583) and 777 (PN777), grown at the Espiguette repository, were selected for maximum phenotypic diversity. These Pinot clones were selected by ENTAV-INRA® in Bourgogne (France) in 1975, 1978 and 1981 for PN386, PN583 and PN777 respectively. PN777 is the clone producing the highest quality wine than PN583 and PN386 [26]. We harvested 5 g of young leaves for nuclear DNA extraction using the NGS method previously described [48]. S-SAP studies were performed on the registered Pinot clones (2 Pinot blanc, 3 Pinot gris, 15 Pinot meunier and 40 Pinot noir) grown in the Espiguette collection. DNA extraction was performed with Qiagen MaxiQKit® according to the manufactory instructions.

### Sequencing samples of PN386, PN583 and PN777 genomes

Approximately 5 µg of nuclear DNA were used for 454 GS-FLX sequencing as previously described [49] at the Genotoul platform (INRA Toulouse Midi-Pyrénées). The data is available from NCBI (FastQ files: SRX098092 for PN386; SRX098091 for PN583 and SRX098090 for PN777). Reads produced using 454 methodology were analyzed with FastQC software (v0.6) developed by Simon Andrews at the Babraham Institute (www.bioinformatics.bbsrc.ac.uk) to validate run quality (sequence number, mean sequence length etc.). We obtained approx. 350 Mb (330–378 Mb) per run, corresponding to approx. one million reads with an average length of 355 bases (Table 5). In terms of base quantity, PN583 was the best run, while both PN777 and PN386 were slightly better in terms of quality (quality score on Phred Sanger graduation [50]). Quality decreased proportionally with read length (Supplementary Figure S3). Duplicated sequences generated by EmPCR bias represented an average of 4974 reads per run (0.58%). There were no overrepresented sequences per run and a very low percentage of contamination by other organisms (132 reads per run on average).

### Aligning PN115, PN386, PN583 and PN777 with the reference genome (PN40024-12X)

We used the Hash-based alignment methods incorporated in the MosaikAssembler tool v1.0 (Wan-Ping Lee and Michael Strömberg, available at bioinformatics.bc.edu/marthlab/). The data set was composed of reads obtained by 454 methodology and PN115 sequences downloaded from NCBI, (Project ID: 18357, www.ncbi.nlm.nih.gov/) [28]. In order to avoid a bias of sequence alignment between the clones studied, the contigs and scaffolds from the PN115 sequences were sheared *in silico* to be considered as data from 454 sequences (size 1000 bases), assuming each nucleotide with optimal quality score.

**Figure 6:**
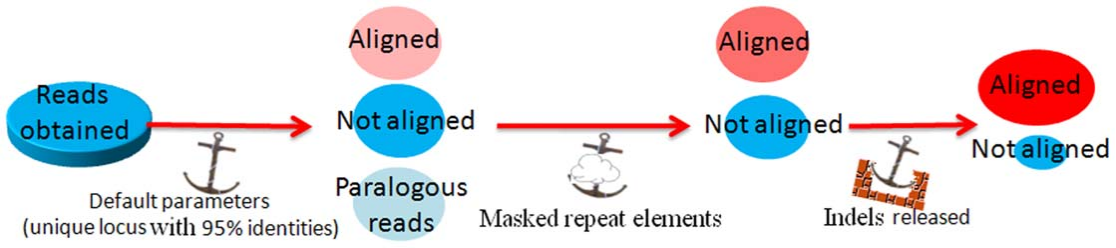
Summary of the alignment method used in the present study. Alignment was accomplished in three successive steps: i) The first alignment used Mosaik with default parameters for 454 GS-FLX: 95% alignment homology in the sequences; ii) Reads not aligned in the first step and that were not paralogs were then filtered with RepeatMasker software. Reads with less than 90% homology with repeat elements were aligned by Mosaik with default parameters. iii) For reads not aligned in the second step, a third alignment was performed using a gap parameter fixed at a minimum (0.1 gap open and extensive penalties).

Sequences of each sample were aligned on the reference genome sequence (PN40024, 12× version (12-Feb 2010)) in three steps: i) alignment of single reads that shared 95% homology with PN40024, ii) unmatched reads were masked for repeat elements and aligned if at least 150 bases were not masked, iii) for the remaining sequences, relaxed stringency was applied with no impact of the gap parameter (Figure 6 and Table 1) (For details on the alignment method, see Methods S1). The origin of non-aligned reads was identified as : i) reads composed of 90% repeat sequences; ii) reads aligned at two loci or more, paralogous reads; iii) reads of cytoplasmic origin (>90% sequence identity with *Vitis vinifera* chloroplast: NC 007957 or mitochondrion: NC 007762); iv) contamination reads originating from other organisms known to be present in laboratories (>90% of identity with *Saccharomyces cerevisiae* S288c (Project ID: 128), *Escherichia coli* 536 (Project ID: 16235), and v) too short (100 pb) or low quality (<Q20) (Mosaik filter) reads (Table 5).

### Polymorphism calling

For all polymorphism calling, identification was first performed *in silico* and all polymorphic loci were then validated manually using EagleView [51]. This manual validation was essential for the following reasons: i) the 454 method is known to create some false positives, particularly with homopolymer sequences ii) the parameters we used for the third alignment (gap parameter fixed at a minimum) may also have created some false positives.

All polymorphisms between 2 clones were called with Gigabayes (http://bioinformatics.bc.edu/marthlab/) between two clones. To reduce false-positive rate, we chose to select polymorphism at a given position, only if a 6.00 fold genome coverage or more was obtained for each clone, and if minority alleles displayed a minimum frequency of 0.3 with an alignment quality score higher than 60 [31]. Polymorphic indels were considered only if they were surrounded by a sequence not localized in the read terminal region and to limit false positives, none of the reads aligned after the third alignment step was used for indel polymorphism detection. A filter was used with RepeatMasker to identify mobile element-linked polymorphisms [52]. Reads composed of a minimum of 150 unmasked bases and a minimum of 100 masked bases were aligned and localized in the reference genome. This polymorphism was called with Gigabayes: indels detected on masked reads were considered as mobile element polymorphisms.

S-SAP was used to validate mobile elements polymorphism as in previously published studies [22], [24], [53] (for details see Methods S1). Primers for retrotransposons were based on sequenced reads containing the LTR region. We chose the most conserved LTR region to design primers in order to amplify the largest transposition loci. A phenetic tree was based on Nei and Li distance matrix [35] from presence/absence data and was built with Darwin software [54] with 1000 permutations (Figure 4).

### Studies of mobile elements activity in the clones' genome

Four mobile elements were analyzed in detail (*Copia10*, *Gret-1*, *Gypsy-19* and *Cauliv-1*). Each insertion generated by these mobile elements was detected and major forms of these element were detected from consensuses form build using AAARF software [55] with the following parameters: 10 LTR reads min, 90% identity. LTR homology sequence trees were obtained using the ClustalW algorithm [56] with 1000 permutations and the neighbor-joining method [57].

## Supporting Information

Figure S1 Percentage of aligned bases with different quality alignment scores. 90% of aligned bases had a quality score of more than 60.
(TIF)

Figure S2 Validation of random distribution of aligned reads. Coefficient correlation between the number of aligned reads and the length of the chromosome was tested using Pearson's correlation (R^2^, P-value<0.05).
(TIF)

Figure S3 Analysis of reads obtained with 454 for each clone using FastQC software. Quality mean per base for each position of base in reads. Quality decreases with length of reads.
(TIF)

Figure S4 The trees in were built from sequence consensus for *Cauliv-1* sequence in 5′LTR. (see Figure 6 in main text). LTR homology sequence trees were obtained using the ClustalW algorithm with 1000 permutations and the neighbor-joining method.
(TIF)

Figure S5 The trees in were built from sequence consensus for *Copia-10* sequence in 5′LTR. (see Figure 6 in main text). LTR homology sequence trees were obtained using the ClustalW algorithm with 1000 permutations and the neighbor-joining method.
(TIF)

Figure S6 The trees in were built from sequence consensus for *Gypsy-19* sequence in 5′LTR. (see Figure 6 in main text). LTR homology sequence trees were obtained using the ClustalW algorithm with 1000 permutations and the neighbor-joining method.
(TIF)

Table S1 Details of polymorphisms detected among clones (SNPs, In/Dels and Mobile elements) with a depth greater than 6× and a base alignment quality score of more than 60 for each of the 3 comparisons.
(DOC)

Table S2 Polymorphisms located in genes between clones. Position corresponds to the beginning of the gene on the genome browser.
(DOC)

Table S3 The first list contains polymorphic mobile elements detected in our data set and ranked by increasing number of polymorphisms. The second list contains mobile elements detected in the sequenced genomes ranked by increasing number of mobile element copies.
(DOC)

Table S4 Results of S-SAP for 4 mobile elements analyzed in detail (*Caul-1, Gret-1, Copia 10, Gypsy 19*). Number of polymorphism bands detected between 2 clones generate by 4 mobile elements analyzed.
(DOC)

Methods S1 We detail in this section the alignment method and S-SAP protocol using in this study.
(DOC)
