# Supplementary figures and images for: Transposable Elements Are a Major Cause of Somatic Polymorphism in Vitis vinifera L
Source: PLoS One. 2012 Mar 12;7(3):e32973. doi: 10.1371/journal.pone.0032973 (PMC3299709; doi:10.1371/journal.pone.0032973)

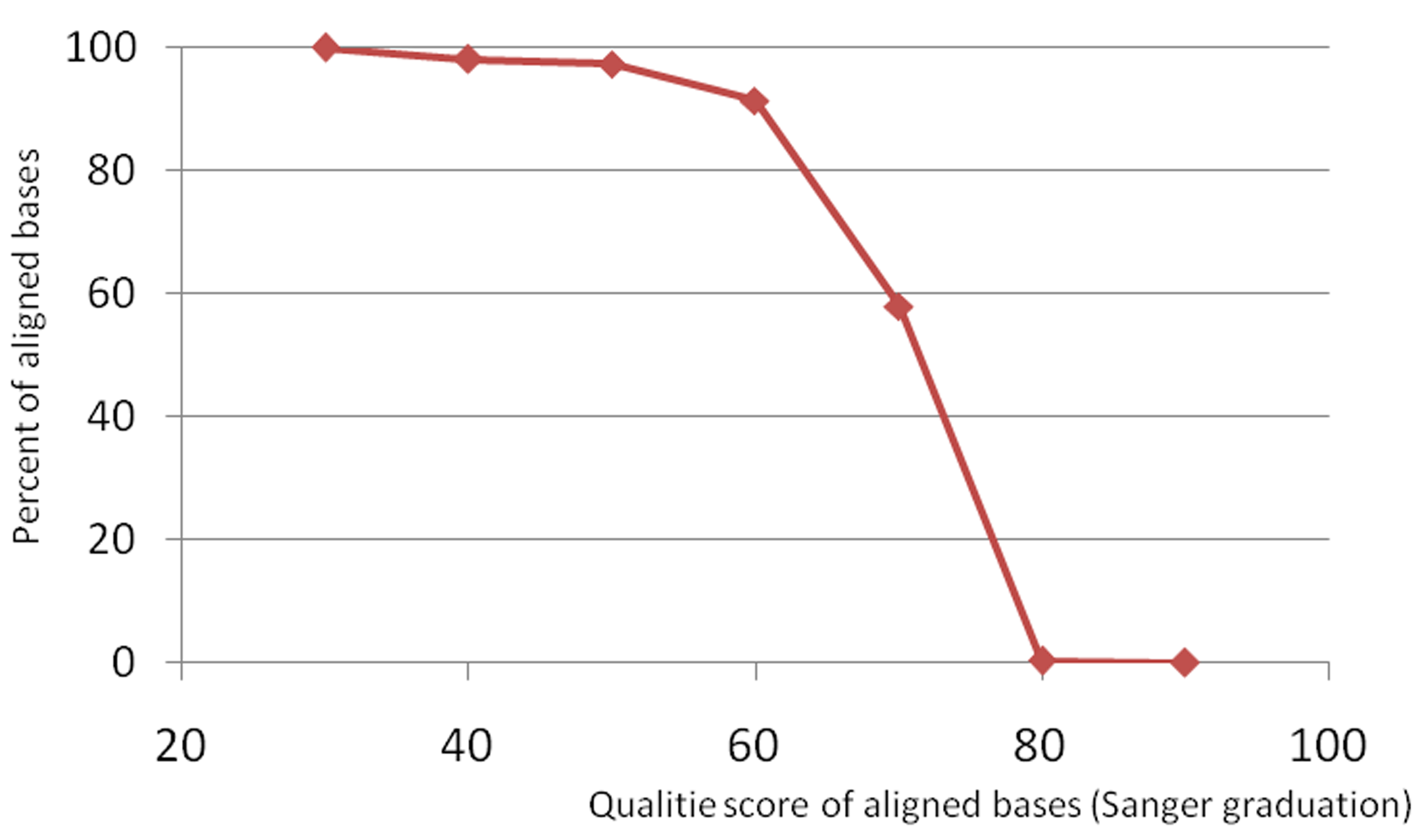

Supplement: Figure S1 — Percentage of aligned bases with different quality alignment scores. 90% of aligned bases had a quality score of more than 60. (TIF) [file pone.0032973.s001.tif]

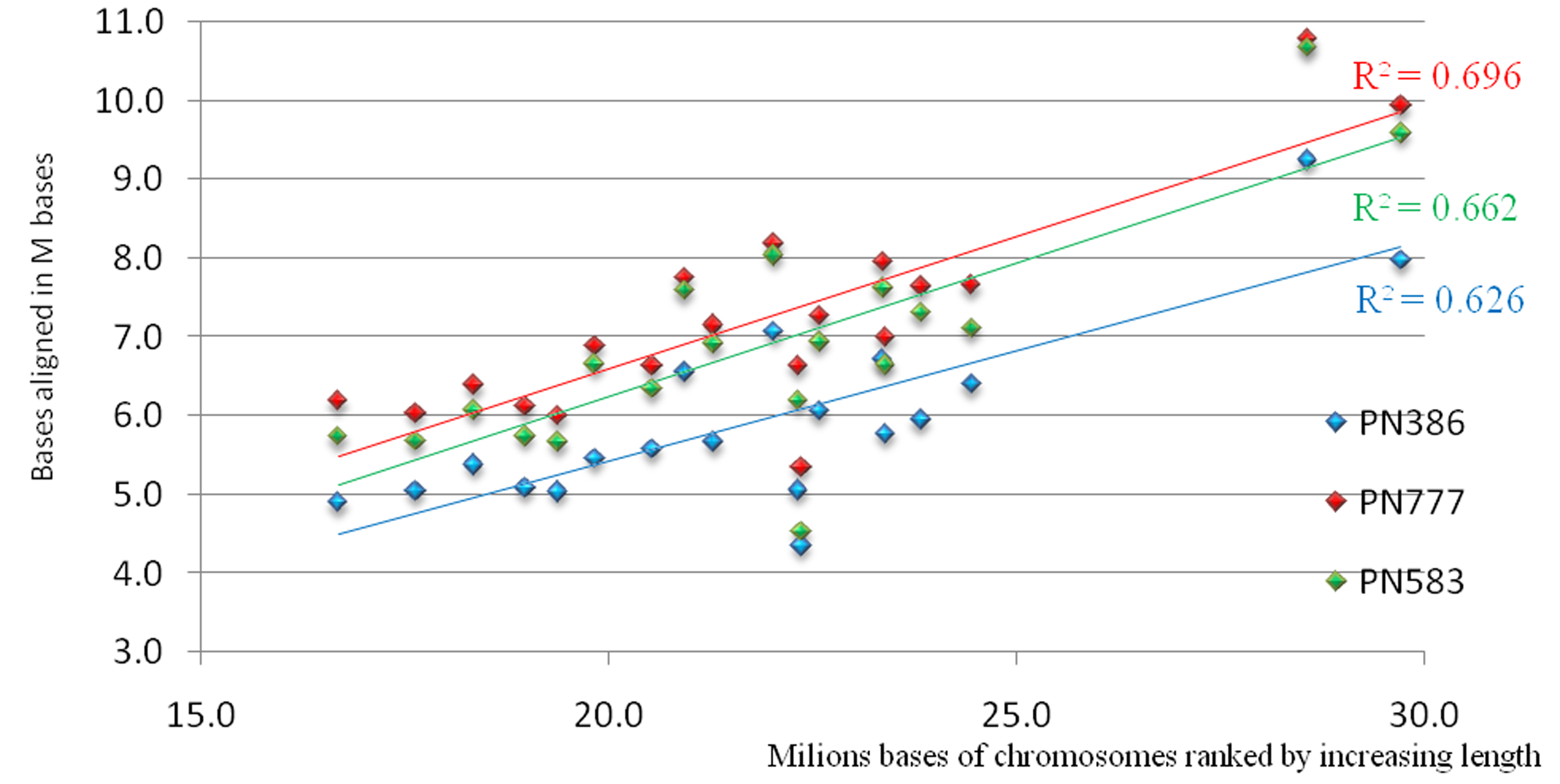

Supplement: Figure S2 — Validation of random distribution of aligned reads. Coefficient correlation between the number of aligned reads and the length of the chromosome was tested using Pearson's correlation (R2, P-value<0.05). (TIF) [file pone.0032973.s002.tif]

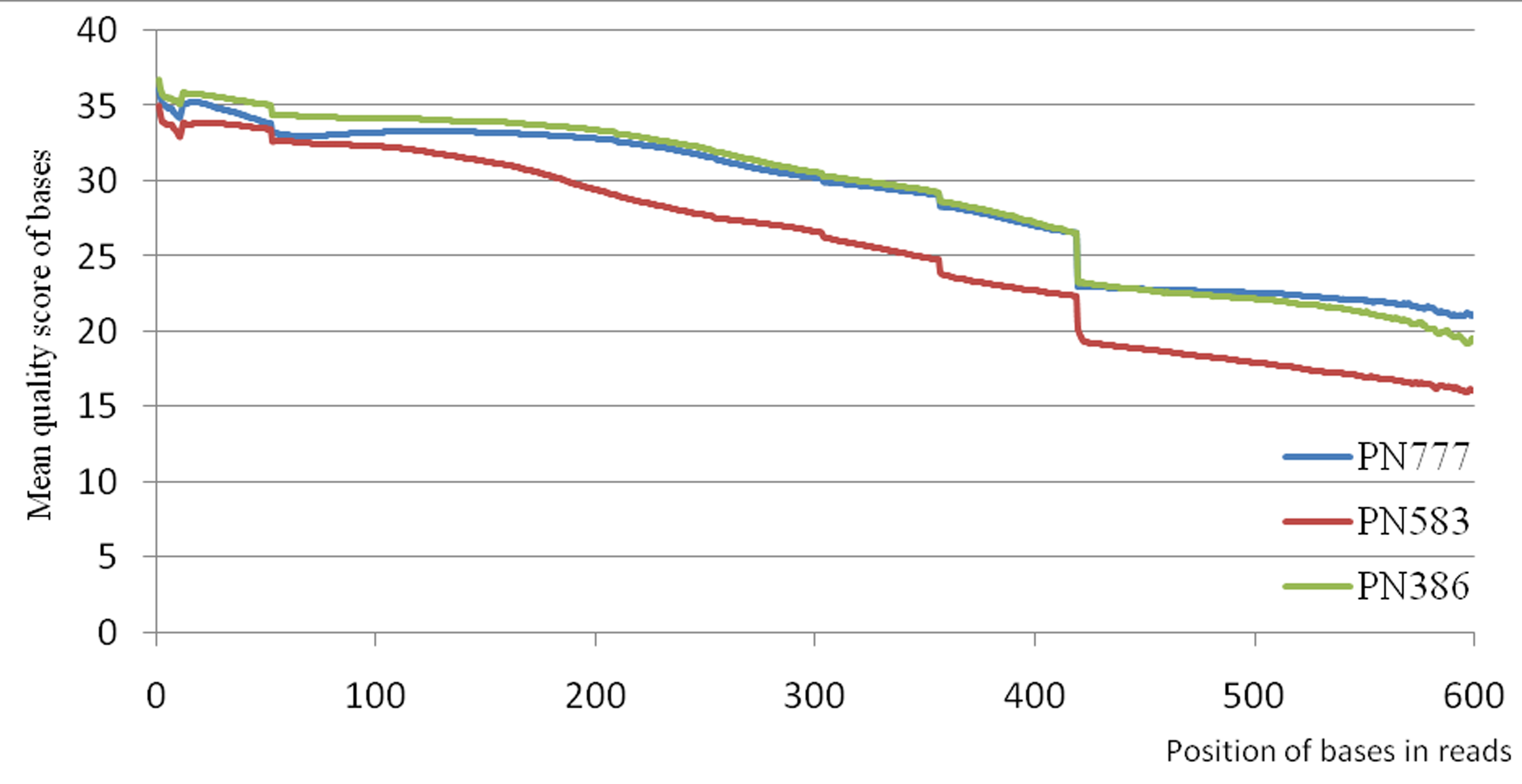

Supplement: Figure S3 — Analysis of reads obtained with 454 for each clone using FastQC software. Quality mean per base for each position of base in reads. Quality decreases with length of reads. (TIF) [file pone.0032973.s003.tif]

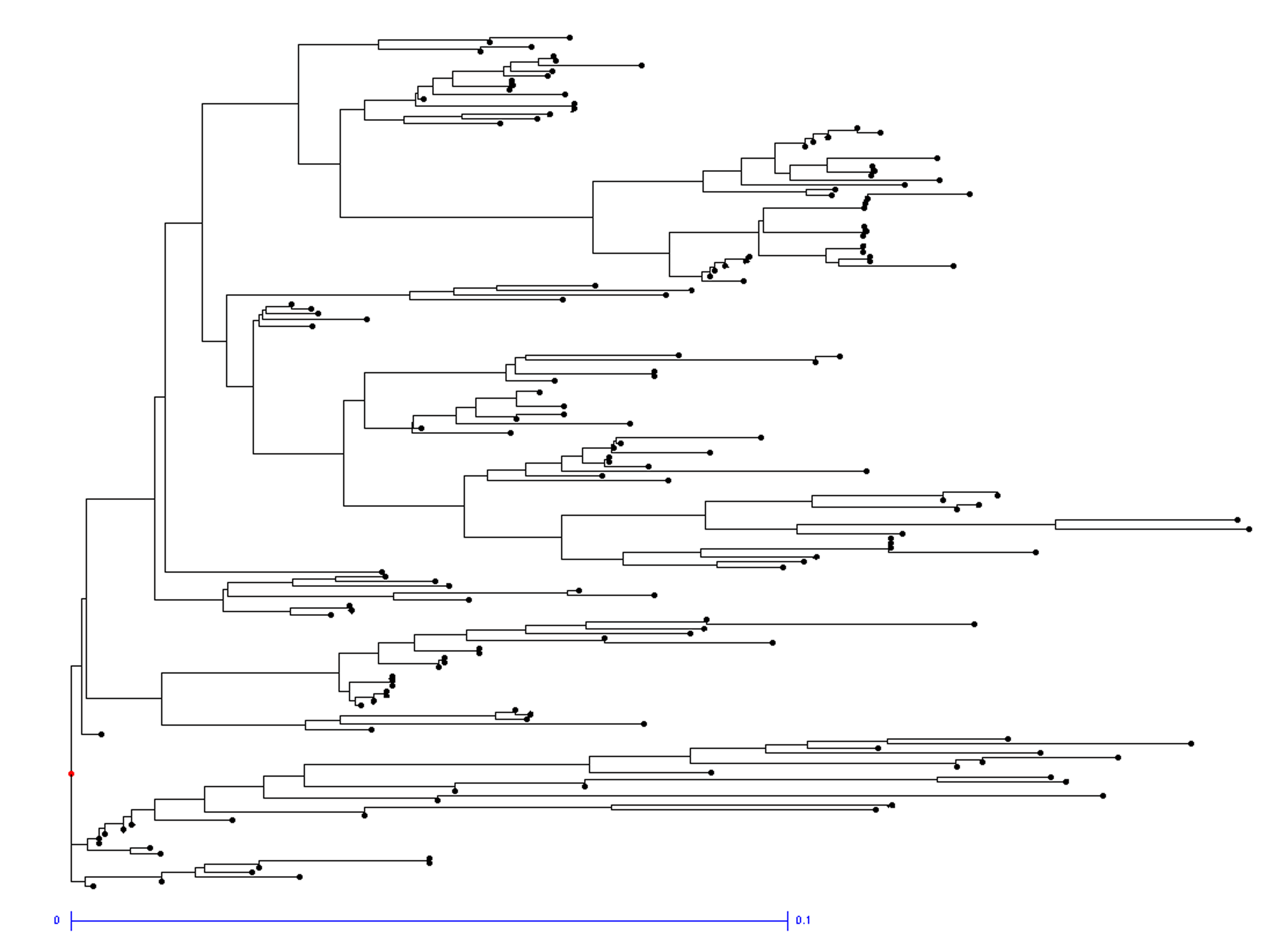

Supplement: Figure S4 — The trees in were built from sequence consensus for Cauliv-1 sequence in 5′LTR. (see Figure 6 in main text). LTR homology sequence trees were obtained using the ClustalW algorithm with 1000 permutations and the neighbor-joining method. (TIF) [file pone.0032973.s004.tif]

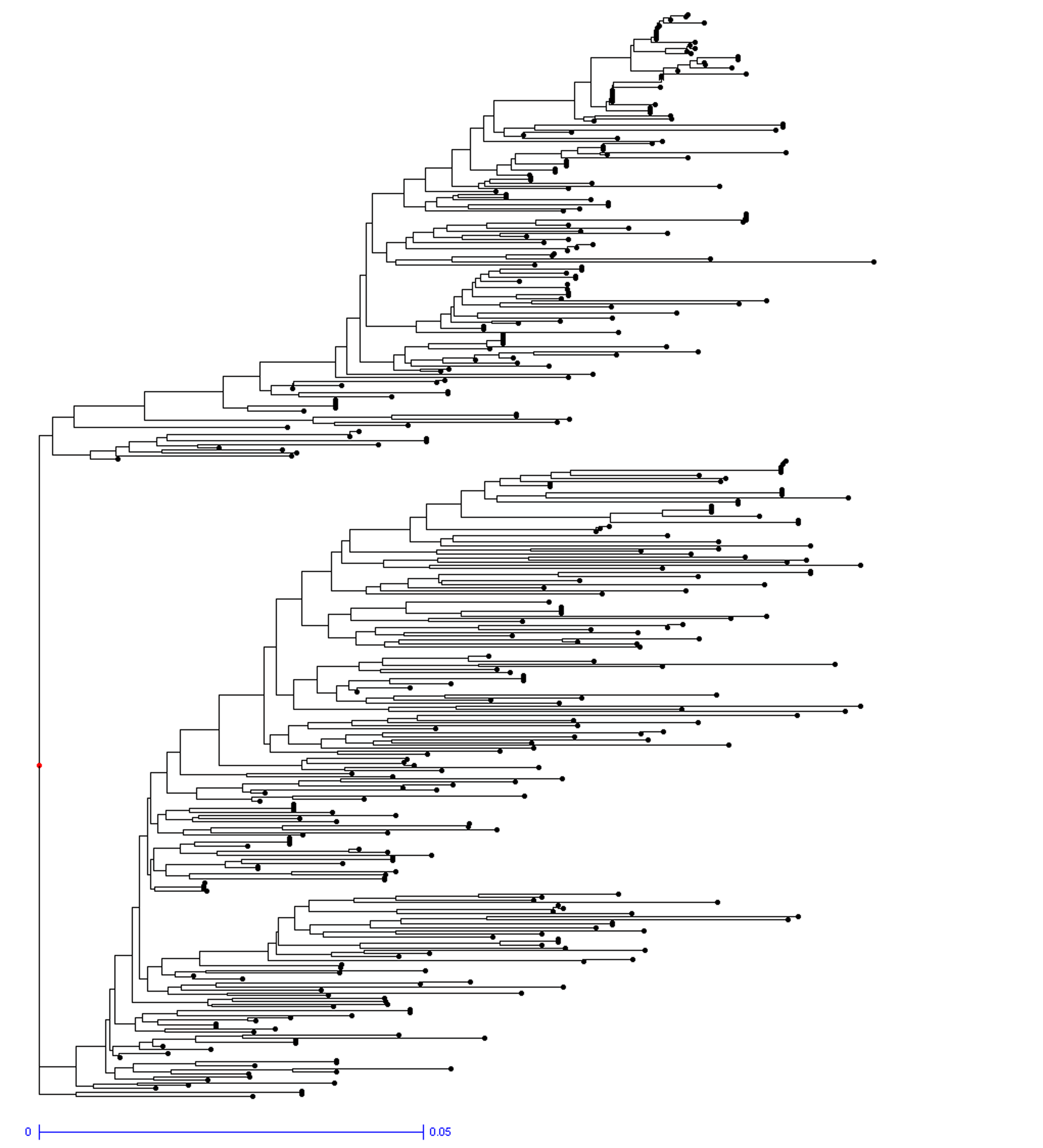

Supplement: Figure S5 — The trees in were built from sequence consensus for Copia-10 sequence in 5′LTR. (see Figure 6 in main text). LTR homology sequence trees were obtained using the ClustalW algorithm with 1000 permutations and the neighbor-joining method. (TIF) [file pone.0032973.s005.tif]

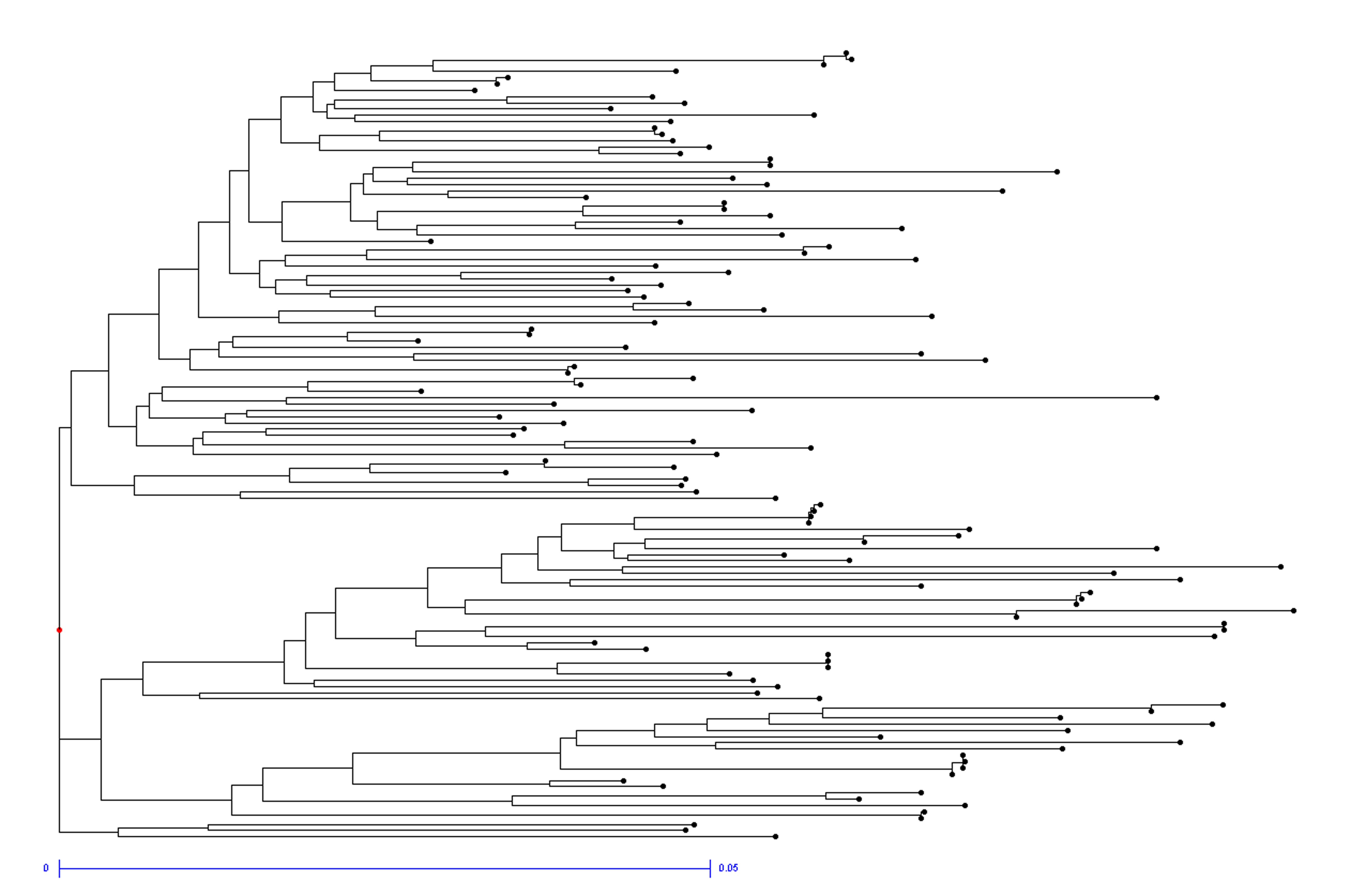

Supplement: Figure S6 — The trees in were built from sequence consensus for Gypsy-19 sequence in 5′LTR. (see Figure 6 in main text). LTR homology sequence trees were obtained using the ClustalW algorithm with 1000 permutations and the neighbor-joining method. (TIF) [file pone.0032973.s006.tif]
