# Supplementary material for: Transposable Elements Are a Major Cause of Somatic Polymorphism in Vitis vinifera L
Source: PLoS One. 2012 Mar 12;7(3):e32973. doi: 10.1371/journal.pone.0032973 (PMC3299709; doi:10.1371/journal.pone.0032973)
Supplement: Table S1 — Details of polymorphisms detected among clones (SNPs, In/Dels and Mobile elements) with a depth greater than 6× and a base alignment quality score of more than 60 for each of the 3 comparisons. (DOC) [file pone.0032973.s007.doc]

|  | |  | |  | | |  |  |  | | | | |
| --- | --- | --- | --- | --- | --- | --- | --- | --- | --- | --- | --- | --- | --- |
| **SNPs** | | PN115 | |  | | | **Mobile el** | PN115 |  | | | | |
| PN777 | | 4 | |  | | | PN777 | 34 |  | | | | |
| PN583 | | 4 | |  | | | PN583 | 75 |  | | | | |
| PN386 | | 11 | |  | | | PN386 | 35 |  | | | | |
| Total | | 19 | |  | | | Total | 147 |  | | | | |
|  | |  | |  | | |  |  |  | | | | |
|  | |  | |  | | |  |  |  | | | | |
| **IN/DEL** | | PN115 | |  | | |  | | | | | | |
| PN777 | | 4 | |  | | |  | | | | | | |
| PN583 | | 1 | |  | | |  | | | | | | |
| PN386 | | 1 | |  | | |  | | | | | | |
| Total | | 6 | |  | | |  | | | | | | |
|  | |  | |  | | |  | | | | | | |
|  |  | |  | |  |  | | | |  |  |  |  |

**Supplementary Table 1.**

Details of polymorphisms detected among clones (SNPs, In/Dels and Mobile elements) with a depth greater than 6X and a base alignment quality score of more than 60 for each of the 3 comparisons.
