# Supplementary material for: Transposable Elements Are a Major Cause of Somatic Polymorphism in Vitis vinifera L
Source: PLoS One. 2012 Mar 12;7(3):e32973. doi: 10.1371/journal.pone.0032973 (PMC3299709; doi:10.1371/journal.pone.0032973)
Supplement: Table S2 — Polymorphisms located in genes between clones. Position corresponds to the beginning of the gene on the genome browser. (DOC) [file pone.0032973.s008.doc]

|  |  |  | |  |  |  | | | |
| --- | --- | --- | --- | --- | --- | --- | --- | --- | --- |
| **Position** | | | **Genes** | | | |  |  |  |
| **Mobiles elements** | | | | | | | Mobiles elements |  |  |
| chr1 | 22892753 | | GSVIVT01001135001 | | | | Gypsy22 |  |  |
| chr1 | 7660970 | | GSVIVT01013782001 | | | | Copia1 |  |  |
| chr10 | 2687291 | | GSVIVT01021220001 | | | | Copia1 |  |  |
| chr10 | 15104708 | | GSVIVT01026261001 | | | | VHARB |  |  |
| chr12 | 6704871 | | GSVIVT01030556001 | | | | Copia23 |  |  |
| chr12 | 21987082 | | GSVIVT01023147001 | | | | VLINE3 |  |  |
| chr13 | 7667083 | | GSVIVT01034686001 | | | | Gypsy12 |  |  |
| chr19 | 1861065 | | GSVIVT01014241001 | | | | Gypsy9 |  |  |
| chr2 | 499949 | | GSVIVT01019417001 | | | | Harbinger_1 |  |  |
| chr2 | 6150229 | | GSVIVT01013259001 | | | | Copia10 |  |  |
| chr5 | 15713361 | | GSVIVT01020995001 | | | | VHARB4 |  |  |
| chr5 | 2568893 | | GSVIVT01017678001 | | | | Gypsy17 |  |  |
| chr5 | 21944397 | | GSVIVT01010735001 | | | | Gypsy22 |  |  |
| chr6 | 13331536 | | GSVIVT01037457001 | | | | Copia3 |  |  |
| chr6 | 14461205 | | GSVIVT01037393001 | | | | Gypsy12 |  |  |
| chr6 | 1440555 | | GSVIVT01025364001 | | | | VLINE1 |  |  |
| chr8 | 13235871 | | GSVIVT01025657001 | | | | Copia22 |  |  |
| chr8 | 2619320 | | GSVIVT01029978001 | | | | Gypsy9 |  |  |
| chr8 | 20392045 | | GSVIVT01033475001 | | | | Gypsy22 |  |  |
| **SNPs** | | | | | | | Located |  |  |
| chr5 | 267707 | | GSVIVT01024255001 | | | | intron |  |  |
| chr12 | 12667503 | | GSVIVT01011544001 | | | | intron |  |  |
| chr13 | 8573986 | | GSVIVT01034732001 | | | | intron |  |  |
| chr18 | 10288579 | | GSVIVT01023526001 | | | | exon - non synonymous - putative peptidase | | |
| chr19 | 4168276 | | GSVIVT01014467001 | | | | intron |  |  |
| **Indels** | | | | | | |  |  |  |
| chr1 | 6979210 | | GSVIVT01000570001 | | | |  |  |  |

**Supplementary Table 2.**

Polymorphisms located in genes between clones. Position corresponds to the beginning of the gene on the genome browser.
