# Supplementary material for: Transposable Elements Are a Major Cause of Somatic Polymorphism in Vitis vinifera L
Source: PLoS One. 2012 Mar 12;7(3):e32973. doi: 10.1371/journal.pone.0032973 (PMC3299709; doi:10.1371/journal.pone.0032973)
Supplement: Table S3 — The first list contains polymorphic mobile elements detected in our data set and ranked by increasing number of polymorphisms. The second list contains mobile elements detected in the sequenced genomes ranked by increasing number of mobile element copies. (DOC) [file pone.0032973.s009.doc]

| **Mobile elements ranked increasing order** | **Polymorphic copies in 4.5Mb of sequenced genomes** |  | **Mobile elements ranked in increasing order** | **Copies of mobile elements from PN386** | **Copies of mobile elements from PN583** | **Copies of mobile elements from PN777** | **mean** |
| --- | --- | --- | --- | --- | --- | --- | --- |
| VLINE3 | 7 |  | VLINE1 | 1796 | 1476 | 1723 | 1665 |
| Gypsy7 | 6 |  | VLINE3 | 1661 | 1246 | 1647 | 1518 |
| Gypsy22 | 6 |  | Copia10 | 1249 | 1236 | 1335 | 1273 |
| Gypsy17 | 5 |  | VLINE2 | 1256 | 1125 | 1288 | 1223 |
| Gret1 | 5 |  | VLINE6 | 1548 | 997 | 977 | 1174 |
| VHARB-N1 | 5 |  | VLINE4 | 1180 | 917 | 1229 | 1109 |
| Gypsy6 | 5 |  | MUDRAVI1 | 933 | 903 | 920 | 919 |
| Copia10 | 4 |  | Gypsy7 | 859 | 894 | 936 | 896 |
| Gypsy12 | 4 |  | Caulimoviridae | 1083 | 889 | 1223 | 1065 |
| Gypsy2 | 4 |  | VHARB-N3 | 1066 | 859 | 1388 | 1104 |
| MUDRAVI2 | 4 |  | VLINE5 | 857 | 824 | 1359 | 1013 |
| VIHAT1 | 4 |  | Gypsy13 | 779 | 809 | 979 | 856 |
| Gypsy3 | 4 |  | VIHAT1 | 785 | 699 | 931 | 805 |
| Gypsy19 | 3 |  | Gypsy17 | 661 | 670 | 782 | 704 |
| Copia-31 | 3 |  | Gypsy12 | 1089 | 668 | 732 | 830 |
| GYVIT1 | 3 |  | MUDRAVI2 | 706 | 659 | 756 | 707 |
| Gypsy9 | 3 |  | MUDRAVI2 | 676 | 657 | 813 | 715 |
| MUDRAVI1 | 3 |  | Gypsy14 | 581 | 586 | 603 | 590 |
| VLINE2 | 3 |  | Gypsy22 | 615 | 547 | 609 | 590 |
| Copia26 | 3 |  | GYVIT1 | 594 | 531 | 630 | 585 |
| VLINE5 | 3 |  | EnSpm-5 | 597 | 509 | 483 | 530 |
| VHARB4 | 3 |  | Gypsy19 | 564 | 490 | 500 | 518 |
| Gypsy14 | 2 |  | VIHAT2 | 467 | 472 | 1068 | 669 |
| Cauliv-1 | 2 |  | MuDR-21 | 455 | 449 | 469 | 458 |
| Gypsy4 | 2 |  | EnSpm-4 | 484 | 431 | 483 | 466 |
| Gypsy13 | 2 |  | Gypsy4 | 422 | 403 | 683 | 503 |
| VLINE1 | 2 |  | Harbinger-3 | 454 | 403 | 489 | 449 |
| Copia22 | 2 |  | MuDR-6 | 367 | 381 | 474 | 407 |
| EnSpm-5 | 2 |  | hAT-10 | 456 | 378 | 397 | 410 |
| VHARB-N3 | 2 |  | MuDR-13 | 526 | 370 | 526 | 474 |
| Harbinger-1 | 2 |  | Gypsy20 | 395 | 350 | 376 | 374 |
| Copia1 | 2 |  | MuDR-8 | 361 | 349 | 407 | 373 |
| VIHAT3 | 2 |  | Gypsy9 | 379 | 340 | 332 | 350 |
| Copia-35 | 2 |  | VHARB4 | 344 | 326 | 349 | 340 |
| Copia15 | 2 |  | ENSPM2 | 421 | 321 | 541 | 427 |
| Copia8 | 2 |  | Gypsy11 | 427 | 314 | 317 | 353 |
| Copia23 | 1 |  | Gypsy18 | 291 | 277 | 276 | 281 |
| Copia11 | 1 |  | MuDR-9 | 301 | 266 | 301 | 289 |
| Gypsy11 | 1 |  | MuDR-9 | 324 | 249 | 279 | 284 |
| Copia17 | 1 |  | Gypsy3 | 260 | 230 | 311 | 267 |
| Harbinger-3 | 1 |  | EnSpm-13 | 239 | 228 | 270 | 246 |
| VLINE6 | 1 |  | MuDR-12 | 266 | 222 | 544 | 344 |
| VLINE4 | 1 |  | VIHAT3 | 209 | 221 | 366 | 265 |
| Copia-32 | 1 |  | Gypsy6 | 220 | 213 | 406 | 279 |
| VIHAT2 | 1 |  | MuDR-18 | 274 | 196 | 209 | 227 |
| MuDR-18 | 1 |  | Harbinger-1 | 200 | 196 | 215 | 204 |
| Copia16 | 1 |  | Gypsy2 | 193 | 189 | 221 | 201 |
| Copia24 | 1 |  | MuDR-4 | 185 | 188 | 166 | 180 |
| Copia27 | 1 |  | EnSpm-3 | 203 | 185 | 178 | 189 |
| hAT-10 | 1 |  | ENSPM-N3 | 207 | 182 | 219 | 202 |
| MuDR-3 | 1 |  | MuDR-3 | 219 | 181 | 186 | 195 |
| Gypsy18 | 1 |  | hAT-6 | 213 | 173 | 279 | 222 |
| Copia25 | 1 |  | ENSPM1 | 187 | 170 | 183 | 180 |
| Copia18A | 1 |  | EnSpm-6 | 183 | 164 | 177 | 175 |
| Copia3 | 1 |  | hAT-7 | 173 | 154 | 179 | 169 |
| EnSpm-3 | 1 |  | VHARB-N2 | 155 | 146 | 155 | 152 |
| Copia19 | 1 |  | hAT-11N | 117 | 114 | 183 | 138 |
| ENSPM-N3 | 1 |  | Vine-1 | 119 | 113 | 169 | 134 |
| hAT-6 | 1 |  | Copia-31 | 109 | 99 | 109 | 106 |
| Copia1A | 1 |  | Gypsy16 | 118 | 92 | 145 | 118 |
| ENSPM1 | 1 |  | MuDR-5 | 87 | 79 | 75 | 80 |
| Copia5 | 1 |  | Copia-33 | 78 | 73 | 147 | 100 |
| VLINE3 | 7 |  | MuDR-11N | 81 | 69 | 75 | 75 |
| Gypsy7 | 6 |  | Gret1 | 64 | 66 | 64 | 65 |
| Gypsy22 | 6 |  | Copia9 | 69 | 51 | 82 | 67 |
| Gypsy17 | 5 |  | Copia26 | 41 | 44 | 117 | 67 |
| Gret1 | 5 |  | Copia23 | 49 | 43 | 49 | 47 |
| VHARB-N1 | 5 |  | Copia-29 | 37 | 35 | 82 | 51 |
| Gypsy6 | 5 |  | Copia22 | 38 | 31 | 50 | 40 |
| Copia10 | 4 |  | Copia17 | 34 | 27 | 29 | 30 |
| Gypsy12 | 4 |  | Helitron1 | 23 | 26 | 32 | 27 |
| Gypsy2 | 4 |  | Copia3 | 19 | 23 | 62 | 35 |
| MUDRAVI2 | 4 |  | Copia11 | 23 | 19 | 17 | 20 |
| VIHAT1 | 4 |  | MuDR-7 | 20 | 19 | 21 | 20 |
| Gypsy3 | 4 |  | Copia12 | 27 | 19 | 17 | 21 |
| Gypsy19 | 3 |  | Copia15 | 21 | 18 | 25 | 21 |
| Copia-31 | 3 |  | Copia5 | 16 | 17 | 25 | 19 |
| GYVIT1 | 3 |  | EnSpm-8N | 17 | 16 | 35 | 23 |
| Gypsy9 | 3 |  | Tvv1 | 18 | 16 | 35 | 23 |
| MUDRAVI1 | 3 |  | Copia18A | 11 | 11 | 15 | 13 |
| VLINE2 | 3 |  | Copia18 | 8 | 10 | 31 | 16 |
| Copia26 | 3 |  | Gypsy1 | 6 | 9 | 9 | 8 |
| VLINE5 | 3 |  | Copia-34 | 7 | 8 | 22 | 12 |
| VHARB4 | 3 |  | Copia-32 | 7 | 8 | 7 | 7 |
| Gypsy14 | 2 |  | Copia1 | 9 | 8 | 22 | 13 |
| Cauliv-1 | 2 |  | Copia16 | 7 | 6 | 19 | 11 |
| Gypsy4 | 2 |  | Copia28 | 3 | 5 | 9 | 6 |
| Gypsy13 | 2 |  | Copia29 | 3 | 5 | 8 | 5 |
| VLINE1 | 2 |  | Copia-35 | 6 | 4 | 14 | 8 |
| Copia22 | 2 |  | Copia7 | 3 | 4 | 9 | 5 |
| EnSpm-5 | 2 |  | Copia24 | 6 | 3 | 8 | 6 |
| VHARB-N3 | 2 |  | Gypsy8 | 3 | 3 | 9 | 5 |
| Harbinger-1 | 2 |  | Copia2 | 2 | 3 | 5 | 3 |
| Copia1 | 2 |  | Copia25 | 3 | 3 | 9 | 5 |
| VIHAT3 | 2 |  | Gypsy15 | 2 | 3 | 5 | 3 |
| Copia-35 | 2 |  | Copia27 | 3 | 2 | 9 | 5 |
| Copia15 | 2 |  | Copia19 | 1 | 2 | 3 | 2 |
| Copia8 | 2 |  | Copia-30 | 2 | 2 | 4 | 3 |
| Copia23 | 1 |  | Copia20 | 2 | 2 | 6 | 3 |
| Copia11 | 1 |  | Gypsy5 | 1 | 1 | 4 | 2 |
| Gypsy11 | 1 |  | Copia6 | 1 | 1 | 4 | 2 |
| Copia17 | 1 |  | Copia1A | 0 | 1 | 2 | 1 |
| Harbinger-3 | 1 |  | Copia13 | 1 | 1 | 2 | 1 |
| VLINE6 | 1 |  | Copia8 | 1 | 1 | 1 | 1 |
| VLINE4 | 1 |  | Gypsy10 | 1 | 1 | 3 | 1 |
| Copia-32 | 1 |  | Copia21 | 1 | 0 | 1 | 1 |
| VIHAT2 | 1 |  | Copia4 | 0 | 1 | 1 | 1 |

**Supplementary Table 3.**

The first list contains polymorphic mobile elements detected in our data set and ranked by increasing number of polymorphisms. The second list contains mobile elements detected in the sequenced genomes ranked by increasing number of mobile element copies.
