# Supplementary material for: Transposable Elements Are a Major Cause of Somatic Polymorphism in Vitis vinifera L
Source: PLoS One. 2012 Mar 12;7(3):e32973. doi: 10.1371/journal.pone.0032973 (PMC3299709; doi:10.1371/journal.pone.0032973)
Supplement: Table S4 — Results of S-SAP for 4 mobile elements analyzed in detail (Caul-1, Gret-1, Copia 10, Gypsy 19). Number of polymorphism bands detected between 2 clones generate by 4 mobile elements analyzed. (DOC) [file pone.0032973.s010.doc]

| **S-SAP** | PN777 | PN583 | PN386 | PN15 |
| --- | --- | --- | --- | --- |
| PN777 |  | 40 | 47 | 41 |
| PN583 |  |  | 58 | 35 |
| PN386 |  |  |  | 51 |
| PN15 |  |  |  |  |

**Supplementary Table 4.**

Results of S-SAP for 4 mobile elements analyzed in detail (*Caul-1, Gret-1, Copia 10, Gypsy 19*). Number of polymorphism bands detected between 2 clones generate by 4 mobile elements analyzed.
