# Supplementary material for: Transposable Elements Are a Major Cause of Somatic Polymorphism in Vitis vinifera L
Source: PLoS One. 2012 Mar 12;7(3):e32973. doi: 10.1371/journal.pone.0032973 (PMC3299709; doi:10.1371/journal.pone.0032973)
Supplement: Methods S1 — We detail in this section the alignment method and S-SAP protocol using in this study. (DOC) [file pone.0032973.s011.doc]

**Detail of alignment method**

We used Has-based alignment methods available in the MosaikAssembler tool v1.0 (Wan-Ping Lee and Michael Strömberg, available at <http://bioinformatics.bc.edu/marthlab/>). The alignment used the reference sequence available at ([http://www.genoscope.cns.fr](http://www.genoscope.cns.fr/), unmasked version dated 12-Feb-2010). The data set comprised reads obtained from 3 Pinot clones in 454 GS-FLX and PN115 sequences produced by Velasco *et al* [1] downloaded from NCBI, Project ID: 18357, <http://www.ncbi.nlm.nih.gov/>). PN115 sequences were processed as 454 sequences, assuming they displayed optimal quality score. Sequences of each sample were aligned on the reference genome sequence (PN40024) in three steps (Figure 1). For the first alignment we used the default parameters provided by MosaikAssembler with 454 GS-FLX Titanium (95% homology with reference sequence and only reads at a single aligned locus). Reads aligned on many loci (paralogous reads) were filtered. In the second alignment, reads that were not aligned in the first step were filtered by RepeatMasker software [2] using library by default and adding the grape mobile element database (<http://www.girinst.org/repbase/>) [3,4]. Reads that were masked but contained a minimum of 150 unmasked bases were aligned by MosaikAssembler with default parameters. The third alignment was performed using a gap parameter fixed at the minimum (0.1 gap open and extensive penalties). We added a tag in order to identify the origin of sample reads and a read for each alignment steps.

Identification of the origin of unmapped reads was accomplished in five classifications: i) reads composed of 90% repeat sequences; ii) reads mapped at two or more loci, paralogous reads; iii) reads of cytoplasmic origin (< 90% of identity with *Vitis vinifera* chloroplast: NC 007957 or *Vitis vinifera* mitochondrion : NC 007762); iv) Contaminated reads from other organisms known to be present in laboratories (< 90% of identity with (Saccharomyces cerevisiae S288c (Project ID: 128), Escherichia coli 536 (Project ID: 16235), and v) bad quality too short reads (100 pb) or bad quality reads (< Q20) (Mosaik filter).

To confirm the correct representation of our aligned data, we compared some criteria (percentage of GC, CnG, CpG and exons) with the reference genome using home perl script and Blast2.0 algorithm [5].

**Details of the S-SAP protocol**

S-SAP was used to validate the polymorphism of mobile elements. To set up this protocol, we referred to previous studies [6,7,8]. DNA extracted from four Pinot noir clones (150 ng) was restricted with MseI (invitroGen). Digestion was performed for 10 h at 65 °C. DNA ligation of *MseI* adapter was prepared by adding (5X) Ligase buffer, 400 U of T4 DNA ligase, 25 pMol of *MseI* adapter and incubated for 4 h at room temperature. T4 Ligase was inactivated by heating at 65 °C for 10 min and samples were stored at 4 °C. Ligated DNA was diluted 1/10 and pre-amplified with 10 mM of *MseI* primer (20 ng DNA, 1X PCR buffer, 3 mM MgCl2, 2.5 mM dNTPs and 1 U Taq DNA polymerase with the following program: one denaturation step at 94°C for 3 min, following by 30 amplification cycles (94 °C for 45 sec, 56 °C for 45 sec, 72 °C for 60 sec) and a final elongation step at 72 °C for 3 min. Selective amplification was performed with 5 µL of pre-amplified DNA, 1X PCR buffer, 3 mM MgCl2, 2.5 mM dNTPs, 10 pM primer of retrotransposon fluorescent markers (FAM or HEX), 10 mM of specific primer of *MseI* at 3 selective bases (1/16 of the genome) (5’ GAT GAG TCC TGA GTA ACG T) and 1U Taq DNA polymerase with the following program: a denaturation step at 94 °C for 3 min, followed by 30 amplification cycles using a touchdown from 65 to 56 °C (94 °C for 45 sec, 65 °Cf or 45 sec with a touchdown of -0.7°C per cycle, 72 °C for 60 sec) followed by 25 amplification cycles (94 °C for 45 sec, 56 °C for 45 sec, 72 °C for 60 sec) and a final elongation step at 72 °C for 3 min. A 1:10 dilution of the fluorescently labeled amplified DNA fragments was run on the Applied Biosystem 3730 xl with the ladder size standard set at 524 bases. The S-SAP profile was analyzed using GeneMapper® (Applied biosystems). We considered there was a peak if the threshold exceeded 150 lux.

**References:**

1. Velasco R, Zharkikh A, Troggio M, Cartwright DA, Cestaro A, et al. (2007) A High Quality Draft Consensus Sequence of the Genome of a Heterozygous Grapevine Variety. PLoS ONE 2: e1326.

2. Chen N (2002) Using RepeatMasker to Identify Repetitive Elements in Genomic Sequences: John Wiley & Sons, Inc.

3. Jurka J, Kapitonov VV, Pavlicek A, Klonowski P, Kohany O, et al. (2005) Repbase Update, a database of eukaryotic repetitive elements. Cytogenetic and Genome Research 110: 462-467.

4. Jurka J, Kapitonov VV, Pavlicek A, Klonowski P, Kohany O, et al. (2000) Repbase Update: a database and an electronic journal of repetitive elements. Trends Genet 9: 418-420.

5. Altschul SF, Gish W, Miller W, Myers EW, Lipman DJ (1990) Basic local alignment search tool. J Mol Biol 215: 403 - 410.

6. Labra M, Imazio S, Grassi F, Rossoni M, Sala F (2004) Vine-1 retrotransposon-based sequence-specific amplified polymorphism for Vitis vinifera L. genotyping. Plant Breeding 123: 180-185.

7. Knox M, Moreau C, Lipscombe J, Baker D, Ellis N (2009) High-throughput retrotransposon-based fluorescent markers: improved information content and allele discrimination. Plant Methods.

8. Wegscheider E, Benjak A, Forneck A (2009) Clonal Variation in Pinot noir Revealed by S-SAP Involving Universal Retrotransposon-Based Sequences. Am J Enol Vitic 60: 104-109.
